# The Impact of Energy Consumption Revolution on Farmers' Happiness: An Empirical Analysis From China

**DOI:** 10.3389/fpubh.2022.778002

**Published:** 2022-03-10

**Authors:** Zhiyao Xu, Rong Ge

**Affiliations:** Institute of Natural Resources and Environmental Audits, School of Government Audit, Nanjing Audit University, Nanjing, China

**Keywords:** happiness, energy consumption, farmer, China, mediation-moderation analysis

## Abstract

This study divided the impact of energy consumption revolution on farmers' happiness into direct and indirect effects. We empirically tested these effects using the Chinese General Social Survey (CGSS) household data in 2015 and the mediation-moderation model. The results showed that: (1) The rural energy consumption revolution has increased the probability of farmers' happiness level by 22.7%. The direct effects with obvious marginal decrement accounted for the main part (over 90%) of the total effect, but the multi-dimensional mediating mechanism was not yet robust. (2) The revolution of rural energy consumption has slightly improved farmers' happiness through the mediating role of increased leisure activities, while the negative impact of increased use-cost on the happiness of low-income farmers was nearly significant. (3) Regional economy, household income, and energy type played negative roles when moderating the above process. To low-income households in the less-developed western region, the total effects were more evident in the aspect of electricity use. Hence, several policy recommendations have been further made, including inclusive energy and strategic synergies.

## Introduction

In response to the Fourth Industrial Revolution, the Chinese government has established the “Energy Consumption Revolution (ECR)” as the essential strategy for China's energy development and formulated concrete plans (1). Specifically, the “ECR” refers to the transition from the traditional energy consumption with intensive emission to the modern energy consumption with low emission (2). However, the Revolution has encountered numerous obstacles in the rural areas of China, the root cause is that Chinese rural population accounts for a high proportion (over 40%) of the total population while sparsely populated throughout China. Whether the Revolution can be successfully implemented in rural China, it depends on not only technological innovations, but also the demand for new energy sources from rural residents. It is crucial to continuously increase rural residents' satisfaction and perception of happiness in the Revolution, so as to motivate their active participation (3).

There is a body of scientific literature on rural energy consumption. First, studies have investigated the Energy Revolution and current status of rural energy consumption. Prior research used energy ladder model, energy stacking model and energy wave model to depict the general course of energy transition (4–6). Some researches described how rural energy consumption evolved from biomass energy (e.g., straw and fuelwood) to the new electrical energy sources [e.g., (4, 7)]. Although the consumption of new energy such as electricity and gas is rapidly growing in China, traditional biomass energy consumption still accounts for more than 60% of total energy consumption in rural China (8, 9). In the rural areas of Beijing, Tianjin and Hebei provinces, fuelwood and coal consumption even accounts for more than 70% of total energy consumption (10). Second, the studies investigated the issue of rural energy supply in China showed, although the commercialization of rural energy is developing fast, due to high costs of energy use, limited technological innovation and financial investment, there are still many challenges in sustainably supplying energy in rural China (11). The urgent problems which need to be solved for rural Energy Reform include the severe pollution from cheap coal energy, the high price and low utilization of clean gas and electricity energy, and the high operating cost of biogas (12). Third, after studying the energy demand in rural China, it is found that household income and the price of energy are the two key factors influencing the demand of rural household energy consumption (13–15). As income level rises, the need for energy upgrade increases for those households (16, 17). It has been proposed that the primary goal of China's energy consumption revolution is to achieve “coal-free” and “fuelwood-free” in the rural areas (2).

Prior research has also explored how energy upgrade impacts the socioeconomic and physical environment, as well as how the socioeconomic and physical environment impacts perceived happiness in rural China. Energy upgrade can significantly reduce the time women spend on housework, on the other hand, increase the time they can spend on leisure activities, increase leisure time and allow rural residents to socialize, entertain, and rest, leading to increased happiness (18). Furthermore, the reduction of traditional energy consumption, such as fuelwood and coal, has greatly decreased the emission of air pollutants such as CO_2_, SO_2_, and NO_x_, which improves the air quality in rural China sharply (15, 19). Improvements for living environment can significantly increase farmers' happiness (20). Thereinto, air quality has been proved playing a significant role when impacting, especially on rural residents' happiness (21). However, there were few studies focus on the relationship between energy consumption and happiness in rural China. Considering the recent energy consumption revolution, this study aimed to quantify the impact of energy consumption on farmers' happiness.

## Mechanism Analysis

**Figure 1:**
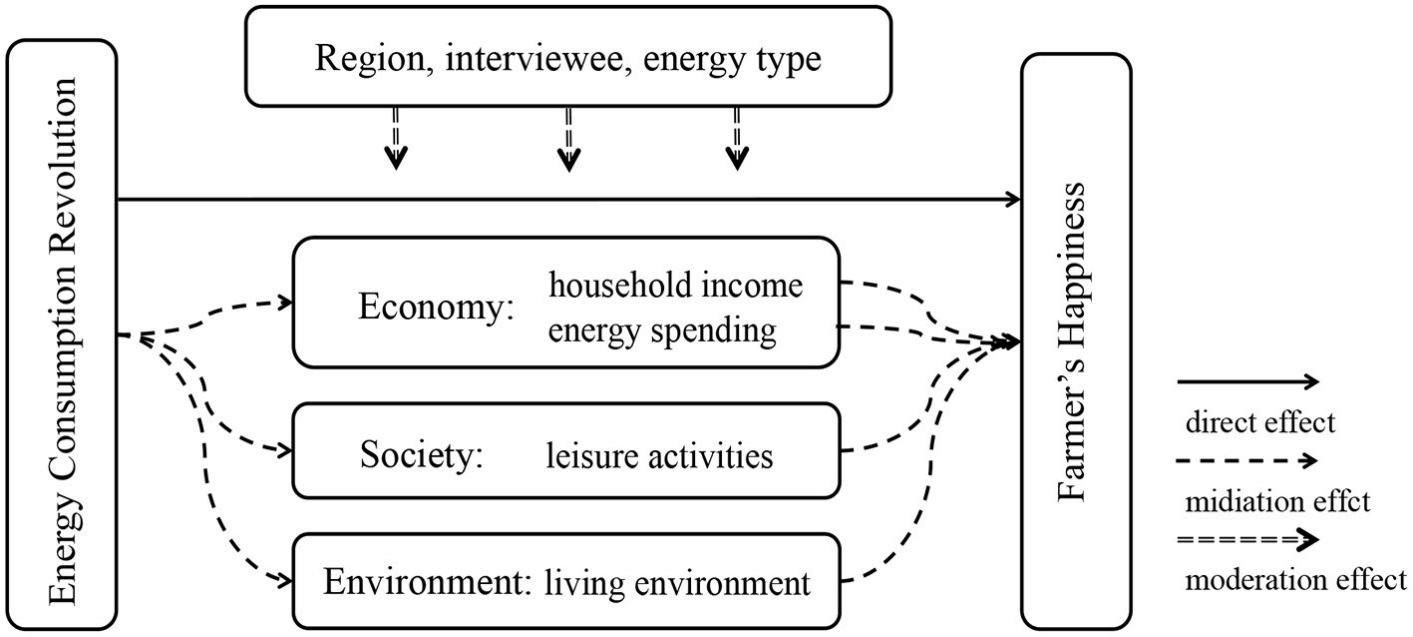
The mechanism of energy consumption revolution effect on farmers' happiness.

Based on the analysis framework of mediation and moderation effects in social psychology, we constructed a conceptual model of the mechanism for energy consumption revolution improving farmers' happiness, as shown in Figure 1. The effects of energy consumption revolution on farmers' happiness include direct effect, mediation effect and moderation effect. Thereinto, the mediation effect consists of three dimensions of economy, society and environment, while the moderation effect contains three dimensionalities: region, economic status of respondents and energy type.

First, the direct effect refers to that the energy consumption revolution directly improves farmers' happiness. On the one hand, using new energy brings rural residents a more comfortable and convenient life, directly increasing people's sense of contentment and happiness; On the other hand, the installation and use of new energy devices bring farmers a strong 'demonstration effect' and pride among neighborhood (22). Although the impact of material consumption on people's happiness is non-linear (23, 24), the marginal effect brought by energy consumption revolution is often positive, especially for rural residents in regions with relatively backward economy (25).

Secondly, the mediation effect refers to that the rural energy consumption revolution indirectly improves farmers' happiness from three dimensions: economy, society and environment. Thereinto, (1) economic dimension refers to that energy consumption revolution enables farmers to devote more time to work and obtain higher income, thus increasing their sense of contentment and happiness (26, 27); meanwhile, the energy consumption revolution may also have negative economic effects, such as resulting in higher energy use costs which will partly neutralize people's happiness. In this paper, household income and electricity cost were used to represent the mediators of these two aspects respectively. (2) The social dimension refers to the fact that the energy consumption revolution liberates farmers from complicated housework and enables them to enjoy more rest, leisure and entertainment time, thus gaining more sense of happiness (28). Here leisure time was used to proxy this mediator variable. (3) The environmental dimension means that the energy consumption revolution can improve the rural living environment and make farmers get more happiness (19). Here people's satisfaction with living environment is used as an intermediary.

Third, some key factors play moderating roles in the process of energy consumption revolution to improve farmers' happiness. (1) People's ability to accept new things differs from regional development levels, so there are certain differences in the mechanism of energy consumption revolution influencing happiness (20, 29). (2) People who are at different socioeconomic levels have significant structural differences in the sources of happiness, and they are various in the process of energy consumption revolution to improve happiness (30). (3) For different energy types, people show different acceptability, and the corresponding effects on people's happiness also vary.

Based on the above analysis, we put forward the following three hypotheses to be tested. (H1) Rural energy consumption revolution can directly improve farmers' happiness. (H2) Rural energy consumption revolution can indirectly improve farmers' happiness through economic, social and environmental dimensions. (H3) Region, people's socioeconomic level and energy type play regulatory roles in the process of consumption revolution to improve happiness.

## Materials and Methods

### Data Source

We analyzed data from the Chinese General Social Survey (CGSS) which started in 2003. Every a few years, the CGSS randomly selects and surveys over 10,000 urban and rural households from all over China. Since 2015, the CGSS questionnaire has added an “Energy Module”, which contains 115 questions related to energy use. In the 2015 CGSS, 10,967 households were interviewed in total. Therein, 3,653 households finished the Energy Module, while 1,472 of them were rural households. In this study, after removing households missing data on essential variables (e.g., happiness, electricity spending), we analyzed data from 1,320 rural households.

### Model Variables

The mediation-moderation models were further applied based on the collected CGSS data. All the involved model variables were listed as follows:

#### Primary Outcome

The primary outcome variable was subjective happiness (*Happ*). The CGSS included the question “Do you think your life is happy?” with five response options: *very unhappy, relatively unhappy, not happy, relatively happy*, and *very happy*. We assigned values 1–5 to the responses, a higher value indicating the greater happiness.

#### Primary Predictor

The primary predictor was the response to the rural energy consumption revolution (*EneRef* ). We defined *EneRef* as the consumption pattern shifting from traditional energy (e.g., fuelwood, straw, and coal) to modern clean energy (e.g., electricity, liquefied gas, natural gas, biogas, and solar energy) (2). If a household has completed the transition to modern energy in at least two of the three activities involving energy consumption (i.e., cooking, showering, and heating/cooling), the household is considered as having responded positively to the national call for energy consumption revolution (*EneRef*_*i*_ = 1). In contrast, if a household does not complete the transition as defined, then *EneRef*_*i*_ = 0 (9).

#### Mediators

The mediators included: (i) annual household income in natural logarithm (*Lginco*), (ii) monthly average electricity spending per person in natural logarithm (*Lgespp*), (iii) leisure activities (*Leis*), and (iv) satisfaction with living environment (*Envir*). Leisure activities were measured using the question “How often do you engage in leisure activities to rest or relax?”, with response options being: *never, rarely, sometimes, often*, and *very often*. Satisfaction with living environment was measured using the question “Are you satisfied with the local government's performance on environmental protection?”, with response options being: *very dissatisfied, dissatisfied, average, satisfied, and very satisfied*.

#### Moderators

The moderators included: (i) geographic region of the household (*Regid*): Eastern, Central, or Western China, (ii) perceived socioeconomic status (*Incox*), and (iii) Energy type (*Enetype*): fuelwood/coal, electricity, liquefied gas/natural gas, or new energy. Perceived socioeconomic status was measured using the question “How do you think about your socioeconomic status by comparing with your peers?”, with response options being: *lower, almost the same, and higher* (20).

#### Covariates

Covariates included participant, household, and regional features. First, we considered nine participant features, consisting gender (*Sex*), age (*Age*), health status (*Heal*), political affiliation (*Poli*), years of education (*Edu*), marital status (*Marr*), employment status (*Work*), religion (*Reli*) and insurance (*Insu*). Therein, health status were categorized into *very unhealthy, relatively unhealthy, average, relatively healthy*, or *very healthy*; political affiliation were categorized into yes for members of the communist party or communist youth league, or no for non-members; marital status were categorized into married or unmarried/divorced/widowed; employment status were categorized into jobless, farmer, or non-farmer; religion were categorized into having any religion or having no religion; and insurance were measured as having each of the four insurance types: basic medical insurance, basic pension, commercial medical insurance, and commercial pension. Second, we considered three household features: quantity of children (*Child*), whether there was a son aged 18–35 (*Son*) (29), and number of houses (*House*). Third, we considered a regional characteristic (*Regn*), which represented the provinces in China.

The descriptive statistics for all the above variables can be found in Table 1.

**Table 1:** Descriptive statistics of the model variables.

**Variable type**	**Variable name**	**Mean**	**Std**	**Min**	**Max**
Outcome variable	Happiness (*Happ*)	3.8689	0.8262	1.0000	5.0000
Primary predictor	Rural energy consumption revolution (*EneRef*)	0.4970	0.5002	0.0000	1.0000
Mediators	Log of household economic income (*Lginco*)	3.2198	1.6160	0.0000	6.9996
	Log of average monthly electricity spending per person (*Lgespp*)	1.3935	0.4217	0.0000	3.3981
	Leisure activities (*Leis*)	3.2303	1.0124	0.0000	5.0000
	Habitat environment satisfaction (*Envir*)	3.4492	0.9604	0.0000	5.0000
Moderators	Region identification (*Regid*)	2.4447	0.96255	1.0000	4.0000
	Socioeconomic status level (*Incox*)	1.6523	0.5516	1.0000	3.0000
	Energy type (*Enetype*)	1.8121	0.4632	1.0000	3.0000
Covariates	Gender of interviewee (*Sex*)	0.4894	0.5001	0.0000	1.0000
	Age of interviewee (*Age*)	52.3909	15.2984	18.0000	91.0000
	Health status (*Heal*)	3.4545	1.1397	1.0000	5.0000
	Political appearance (*Poli*)	0.0818	0.2742	0.0000	1.0000
	Years of education (*Edu*)	6.5508	4.0692	0.0000	19.0000
	Marital status (*Marr*)	0.8265	0.3788	0.0000	1.0000
	Work status (*Work*)	0.8811	0.7287	0.0000	2.0000
	Religion (*Reli*)	0.1174	0.3220	0.0000	1.0000
	Number of children (*Child*)	2.1144	1.3426	0.0000	10.0000
	Son aged 18–35 (*Son*)	0.2492	0.4327	0.0000	1.0000
	Number of properties (*House*)	1.1212	0.4445	0.0000	5.0000
	Social insurance (*Insu*)	1.6788	0.6550	0.0000	4.0000

*The statistics were based on the China General Social Survey (CGSS) in 2015*.

### Model Analysis

We conducted data analysis in three steps using STATA version 16. First, using subjective happiness as the outcome, we built benchmark regression models, take response to the rural energy consumption revolution and the covariates as the predictors (20, 29–31).


(1) $$\begin{array}{l} Happ_{i}=Ctem+\alpha_{xy}EneRef_{i}+\sum \gamma_{m}Cont_{mi}+\theta Regn_{j}+\varepsilon_{i} \end{array}$$

In Equation (1), *Happ*_*i*_ is the *i*_th_ household's subjective happiness, *EneRef*_*i*_ is the binary variable indicating whether the farmer responded positively to the energy consumption revolution, *Cont*_*mi*_ is a series of covariates including participant and household characteristics, *Regn*_*j*_ is the fixed effect for the *j*_th_ province, *Ctem* is a constant, and ε_*i*_ is the random error.

Second, to inspect potential mediation effects, we built mediation models by adding mediators into the above benchmark model as follows:


(2) $$\begin{array}{l} Medi_{i}=Ctem+\alpha_{xz}EneRef_{i}+\sum^{\text{​}}\gamma_{m}Cont_{mi}\\ \;+\theta Regn_{j}+\varepsilon_{i} \end{array}$$

(3) $$\begin{array}{l} Happ_{i}=Ctem+\alpha_{xy^{\prime }}EneRef_{i}+\alpha_{zy}Medi_{i}+\sum^{\text{​}}\gamma_{m}Cont_{mi}\\ \;+\theta Regn_{j}+\varepsilon_{i} \end{array}$$

In Equations (2) and (3), *Medi* is the potential mediators (i.e., *Lginco, Lgespp, Leis* or *Envir*). Since *Happ, Leis* and *Envir* are ordinal variables, we modeled these variables with ordered probability model. The other outcomes were modeled with linear regression based on the Ordinary Least Squares (OLS) method.

When using the ordered probability model in mediation analysis, the decomposition of total effect based on the traditional linear regression is not applicable. Therefore, we adopted the modified mediation effect test and decomposition method as described in (32, 33). In this method, the first step is to conduct a coefficient *t*-test of rural energy consumption revolution (*EneRef* ) in the benchmark model (Equation 1). If α_*xy*_ is significant, we proceed to the second step; otherwise, the mediation effect is considered to be non-significant. The second step is to test models in Equation (2) and (3) separately. If the *t*-tests for both α_*xz*_ and α_*zy*_ are significant, we would skip third step and move forward to the fourth step; otherwise, we would proceed the third step to conduct an Iacobucci-z test:


(4) $$\begin{array}{l} z=\frac{z_{\alpha_{xz}}z_{\alpha_{zy}}}{\sqrt{z_{\alpha_{xz}}^{2}+z_{\alpha_{zy}}^{2}+1}} \end{array}$$

In Equation (4), *z*_*xz*_ and *z*_*zy*_ are the *t*-statistics of α_*xz*_ and α_*zy*_ in regression models 2 and 3. If the test results for Iacobucci-z is not significant, the mediation effect will be non-significant. If the Iacobucci-z test is significant, we proceed to the fourth step, in which we will determine whether the *t*-test of $\alpha_{xy^{\prime }}$ is significant or not. If the *t*-test is non-significant, it will be a complete mediation effect. In contrast, if there is significant *t*-test, then it indicates a partial mediation effect and the Breen decomposition must be used to calculate the direct and indirect effects in the fifth step (33). In step 5, we define σ_*e*_=*sqrt*(3)σ_ε_/π, where σ_ε_ is the standard error of the random error ε_*i*_ in Equation (3), the direct effect is $\alpha_{\text{x}{\text{y}}^{\text{′}}}$/σ_*e*_, the mediation effect is α_*xz*_α_*zy*_/σ_*e*_, and the total effect is ($\alpha_{xy^{\prime }+}$Σα_*xz*_α_*zy*_)/σ_*e*_.

Lastly, we built separate mediation models stratified by the moderators (i.e., *Regid, Incox*, and *Enetype*).

## Results

### Respondents' Characteristics

**Figure 2:**
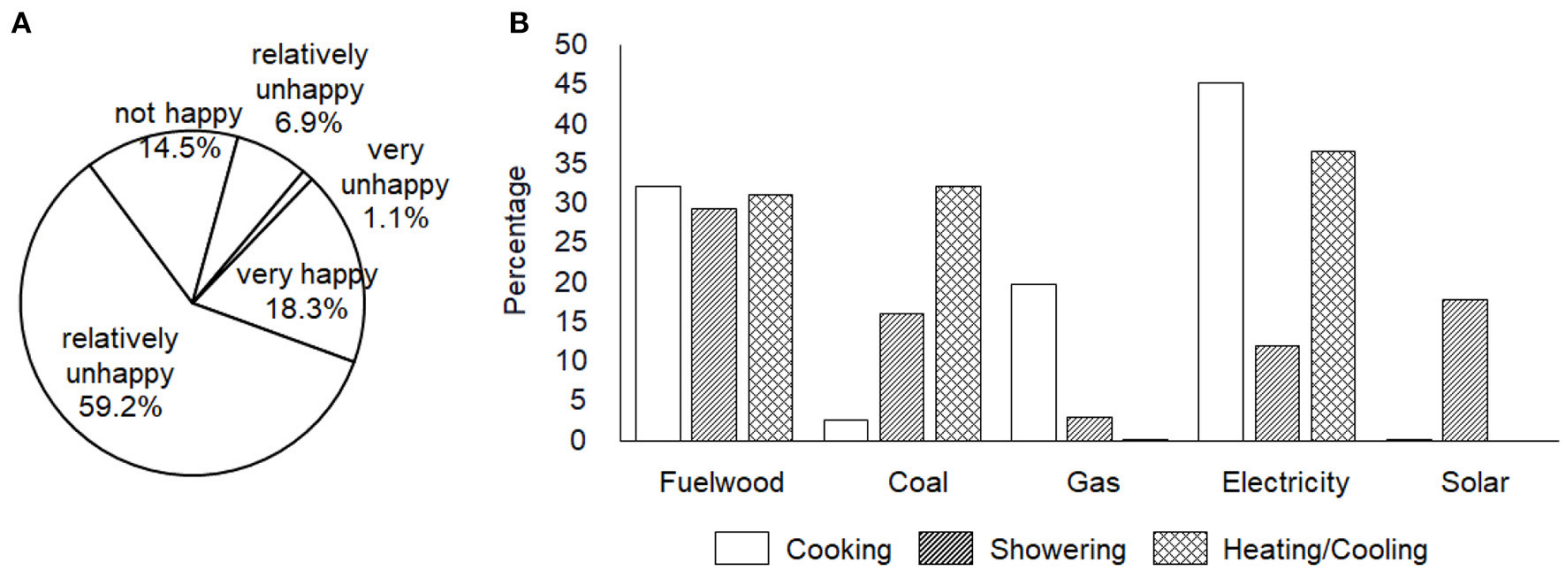
The data distribution of happiness index **(A)** and energy consumption revolution **(B)** based on the China General Social Survey (CGSS) in 2015.

In the 1320 rural households included in this analysis, there were respectively, 14 (1.1%), 91 (6.9%), 191 (14.5%), 782 (59.2%), and 242 (18.3%) households chose “*very unhappy*”, “*relatively unhappy*”, “*Just so-so*”, “*relatively happy*” and “*very happy*” (Figure 2A). In cooking activity, 35% of the total households remained to use fuelwood and coal, while 45% and 20% chose electricity and gas, separately; for showering, 45% still used fuelwood and coal, while 18% and 12% used solar and electricity, separately; for heating/cooling, 63% still kept fuelwood and coal, while 37% used electricity (Figure 2B). Overall, a total of 656 (49.7%) households responded positively to the call for rural energy consumption revolution through various measures. However, only 23.5% of the 1,320 households completed the structural transition to modern energy in all of the three main energy consumption activities.

### Benchmark Regression Models

We summarized the results from benchmark regression models in Table 2. Regression model 1 shows that, responding proactively to the energy consumption revolution may increase the probability of enhancing farmers' happiness level by 22.7%. The regression results for (2–1), (2–2), (2–3) and (2–4) of potential mediating variables *Lginco, Lgespp, Rest* and *Envir*, showed that the rural energy consumption revolution did not significantly increase household income, while it significantly increased per capita electricity expenditure and also the rest time of farmers. Likewise, it was inapparent in the impact on the satisfaction of rural living environment. Furthermore, regression models 1 and 3 show that older age, better health status, and possessing more house property were significantly associated with increasing of happiness, while having a son aged 18–35 was significantly associated with decreasing of happiness. It is not observed any significant association among happiness and other covariates including sex, political affiliation, years of education, marital status, employment status, religion, number of children in the household, and insurance.

**Table 2:** Benchmark regression results of the mediation model.

	***Happ***	***Lginco***	***Lgespp***	***Leis***	***Envir***	***Happ***
	**(Model 1)**	**(Model 2–1)**	**(Model 2–2)**	**(Model 2–3)**	**(Model 2–4)**	**(Model 3)**
*EneRef*	0.227^***^	0.085	0.130^***^	0.196^***^	0.071	0.211^***^
	(0.070)	(0.086)	(0.025)	(0.065)	(0.067)	(0.073)
*Lginco*			0.004	−0.029	−0.019	−0.008
			(0.008)	(0.022)	(0.022)	(0.022)
*Lgespp*		0.043		−0.105	−0.076	−0.035
		(0.096)		(0.070)	(0.075)	(0.084)
*Leis*		−0.056	−0.016		0.002	0.088^***^
		(0.043)	(0.011)		(0.033)	(0.034)
*Envir*		−0.033	−0.012	0.003		0.089^**^
		(0.043)	(0.012)	(0.033)		(0.036)
*Sex*	−0.108	0.546^***^	−0.010	0.050	0.034	−0.112
	(0.068)	(0.085)	(0.025)	(0.064)	(0.063)	(0.069)
*Age*	0.009^***^	0.006	0.002^*^	−0.001	0.010^***^	0.009^***^
	(0.003)	(0.004)	(0.001)	(0.003)	(0.003)	(0.003)
*Heal*	0.261^***^	0.151^***^	−0.008	0.047	0.017	0.260^***^
	(0.034)	(0.039)	(0.011)	(0.030)	(0.032)	(0.034)
*Poli*	0.155	0.128	0.113^**^	0.154	0.009	0.156
	(0.112)	(0.140)	(0.049)	(0.106)	(0.104)	(0.117)
*Edu*	0.013	0.007	0.006^*^	0.030^***^	−0.000	0.012
	(0.010)	(0.013)	(0.003)	(0.010)	(0.010)	(0.011)
*Marr*	−0.004	0.492^***^	−0.066^*^	−0.119	0.098	−0.016
	(0.091)	(0.121)	(0.035)	(0.085)	(0.087)	(0.092)
*Work*	−0.078	0.787^***^	0.044^**^	−0.128^***^	0.030	−0.055
	(0.048)	(0.068)	(0.018)	(0.048)	(0.049)	(0.051)
*Reli*	0.142	0.060	0.040	0.183^*^	−0.092	0.160
	(0.100)	(0.129)	(0.040)	(0.108)	(0.104)	(0.102)
*Child*	0.046	−0.025	−0.008	0.057^*^	−0.014	0.044
	(0.032)	(0.039)	(0.011)	(0.031)	(0.034)	(0.032)
*Son*	−0.212^***^	−0.073	0.038	−0.051	0.085	−0.213^***^
	(0.076)	(0.092)	(0.027)	(0.069)	(0.073)	(0.077)
*House*	0.218^**^	0.243^***^	0.032	0.081	−0.044	0.227^***^
	(0.086)	(0.081)	(0.025)	(0.066)	(0.075)	(0.088)
*Insu*	0.019	0.116^*^	0.025	0.058	0.021	0.017
	(0.048)	(0.065)	(0.017)	(0.045)	(0.045)	(0.049)
*Fixed effect*	Y	Y	Y	Y	Y	Y
*Sample size*	1320	1320	1320	1320	1320	1320
R^2^	0.071	0.261	0.100	0.042	0.021	0.091

***, **, and * *indicate that the results are significant at 1, 5, and 10% levels, respectively*.

### Mediation Analysis

We summarized results from the mediation models in Table 3. As showed in the table, the tests of α_*xy*_, α_*xz*_, α_*zy*_ and $\alpha_{xy^{\prime }}$ were *t*-test statics about coefficient of rural energy consumption revolution (*EneRef* ) in the benchmark models 1, 2, and 3. The Iacobucci-z test was used to confirm the significance when only one of the α_*xz*_ and α_*zy*_ was significant. In the first step, α_*xy*_ test was significantly positive for all mediators. In the second step, the α_*xz*_ test was significantly positive for electricity expenditure (*Lgespp*) and leisure activities (*Leis*), but non-significant for household income (*Lginco*) and living environment satisfaction (*Envir*). In the third step, the α_*zy*_ test is significantly positive for leisure activities (*Leis*) and living environment satisfaction (*Envir*), but non-significant for household income (*Lginco*) and electricity spending (*Lgespp*). In the fourth step, the Iacobucci-z test showed that the mediation effects of electricity spending (*Lgespp*) and living environment satisfaction (*Envir*) were not significant. In the fifth step, the $\alpha_{xy^{\prime }}$ test revealed that only leisure activities (*Leis*) were a significant mediator of the relationship between energy consumption revolution and farmers' happiness. The other potential mediators were not significant.

**Table 3:** Testing results and decomposition of the mediation effect of energy consumption revolution on improving farmers' happiness.

	**Potential mediators**	**Test of *α_xy_***	**Test of *α_*xz*_***	**Test of α_zy_**	**Iacob-z test**	$\alpha_{xy^{\prime }}$ **test** **(direct effect)**	**Mediation effect**	**Total effect**
Economic effect	*Lginco*	0.227^***^	0.085	−0.008	-		Non- significant	
		(0.070)	(0.086)	(0.022)				
	*Lgespp*	0.227^***^	0.130^***^	−0.035	−0.005 (−0.011)		Non-significant	
		(0.070)	(0.025)	(0.084)				
Social effect	*Leis*	0.227^***^	0.196^***^	0.088^***^	-	0.211^***^	0.017^*^	0.227^***^
		(0.070)	(0.065)	(0.034)		(0.073)	(0.009)	(0.070)
Environmental effect	*Envir*	0.227^***^	0.071	0.089^**^	0.006 (0.006)		Non-significant	
		(0.070)	(0.067)	(0.036)				

*All regressions include all control variables and fixed effects*;

***, **, and * *indicate that the regression results are significant at 1, 5, and 10% levels, respectively*.

We further applied the Breen decomposition to quantify the mediation effects. As seen in Table 3, in the relationship where rural energy consumption revolution significantly increased farmers' happiness the direct effect accounted for over 90% of the total effect and the mediation effect accounted for <10% of the total effect. All of the mediation effects were brought by increased leisure activities (*Leis*), while the mediation effect of economic [i.e., household income (*Lginco*) and electricity spending (*Lgespp*)] and environmental (*Envir*) factors was not significant.

### Moderation Analysis

We summarized results from the moderation analysis in Table 4. As seen in the table, the effect of energy consumption revolution on farmers' happiness varied slightly across geographic regions. Overall, the direct effect of energy consumption revolution on increasing farmers' happiness decreased from the western to central-eastern regions, with the largest value being observed in the less-developed western region. Besides, farmers in the more-developed central-eastern region are more likely to prefer leisure activities when they have time. So, the leisure activities were significantly increased because of the energy consumption revolution in central-eastern China, which in turn significantly increased farmers' happiness.

**Table 4:** The moderation effect of energy consumption revolution on improving farmers' happiness.

	**Group**	**Gross effect**	**Direct effect**	**Indirect effect**	
Region (*Regid*)	Western regions	0.274^**^ (0.118)	0.248^**^ (0.121)	*Lginco*	Non-significant
				*Lgespp*	Non-significant
				*Leis*	Non-significant
				*Envir*	Non-significant
	Central-eastern regions	0.231^***^ (0.087)	0.209^**^ (0.089)	*Lginco*	Non-significant
				*Lgespp*	Non-significant
				*Leis*	significant(+)
				*Envir*	Non-significant
Income level (*Incox*)	Low income	0.238^**^ (0.116)	0.274^**^ (0.118)	*Lginco*	Non-significant
				*Lgespp*	Near-significant(-)
				*Leis*	Non-significant
				*Envir*	Non-significant
	High income	0.177^*^ (0.090)	0.142 (0.092)	*Lginco*	Non-significant
				*Lgespp*	Non-significant
				*Leis*	Significant(+)
				*Envir*	Non-significant
Energy type (*Enetype*)	Electricity	0.217^**^ (0.085)	0.206^**^ (0.087)	*Lginco*	Non-significant
				*Lgespp*	Non-significant
				*Leis*	Significant(+)
				*Envir*	Non-significant
	Gas (liquefied gas/natural gas)	0.267 (0.207)	0.221 (0.220)	*Lginco*	Non-significant
				*Lgespp*	Non-significant
				*Leis*	Non-significant
				*Envir*	Non-significant
	New energy	0.130 (0.213)	0.109 (0.228)	*Lginco*	Non-significant
				*Lgespp*	Non-significant
				*Leis*	non-significant
				*Envir*	Non-significant

*All regressions include all control variables and fixed effects*;

***, ** and * *indicate that the regression results are significant at 1, 5, and 10% levels, respectively*.

Table 4 reported the moderation effect of household income (*Incox*) on the relationship between energy consumption revolution and farmers' happiness. We observed large differences in the effect of the energy consumption revolution on happiness across different household income levels. First, the direct effect of energy consumption revolution on happiness decreased from the low-income households to middle and high-income households. Second, farmers from middle- and high-income households cared more about leisure activities. The energy consumption revolution significantly increased the happiness of middle- and high-income farmers by increasing leisure activities, but the mediation effects of economic and environmental factors were not significant. Third, low-income farmers were more susceptible to the rising cost of clean energy. The energy consumption revolution near significantly reduced the happiness of low-income farmers because of the increasing electricity spending by energy consumption revolution.

The moderation effect of energy type (*Enetype*) was also reported in Table 4. Different types of energy had differential effects on farmers' happiness. First, the total effect of electricity, gas, and new energy on increasing farmers' happiness decreased in this order, with only electricity being significant. Second, electrical energy consumption significantly increased farmers' happiness by increasing leisure activities, while the effect of new energy sources such as liquefied gas, natural gas and especially solar energy was not significant.

### Endogenous Treatment and Robustness Test

Endogenesis may come from a variety of complex factors, which can lead to systematic bias in estimates. As the model was based on cross-sectional data, propensity score matching (PSM) method was adopted to deal with the endogeneity problem (34). According to all control variables, 656 households were matched with 1:1 nearest neighbor, and 1312 matched regression samples were obtained. We found that there was a significant difference in density distribution between the control group and the treatment group before matching, and the density distribution of the control group was closer to that of the treatment group from all dimensions after matching, so the “systematic bias” between the control group and the treatment group could be better eliminated, and the propensity score matching achieved a good effect.

Further, we did a re-regression of the above benchmark and mediation model based on matched samples (Table 5). In terms of the magnitude, direction and significance of the key coefficients,the regression results before and after matching were consistent. Therefore, it is believed that there is no obvious bias in our models and the endogeneity problem will not have a systematic impact on the regression model.

**Table 5:** Regression results of benchmark model and mediation model after propensity score matching.

	***Happ***	***Lginco***	***Lgespp***	***Rest***	***Envir***	***Happ***
*EneRef*	0.233^***^	0.075	0.126^***^	0.187^***^	0.079	0.218^***^
	(0.071)	(0.087)	(0.025)	(0.065)	(0.068)	(0.072)
*Lginco*			0.004	−0.029	−0.018	−0.001
			(0.008)	(0.022)	(0.022)	(0.022)
*Lgespp*		0.043		−0.107	−0.071	−0.015
		(0.097)		(0.071)	(0.076)	(0.084)
*Rest*		−0.056	−0.016		0.008	0.087^**^
		(0.043)	(0.011)		(0.033)	(0.034)
*Envir*		−0.030	−0.012	0.009		0.101^***^
		(0.043)	(0.012)	(0.034)		(0.036)
*Samples*	1312	1312	1312	1312	1312	1312
R^2^	0.073	0.261	0.101	0.044	0.023	0.091

*All regressions include all control variables and fixed effects*;

*** and ** *indicate that the regression results are significant at 1 and 5% levels, respectively*.

**Figure 3:**
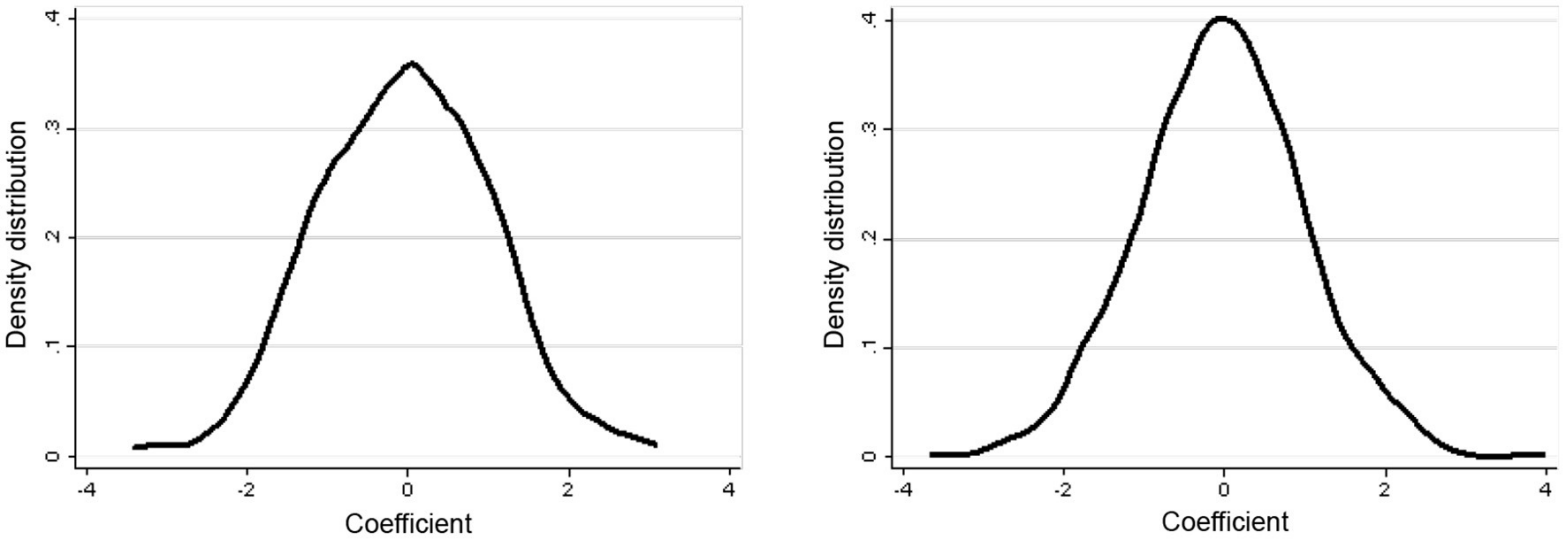
Placebo test results using 500 and 1,000 random counterfactual explanatory variables. All regressions include all control variables and provincial fixed effects.

Also, we did a serial of robustness tests. First, we adjusted the definition of Energy consumption Revolution from “If a household *i* has completed at least two of the three activities involving energy consumption, then *EneRef*_*i*_ = 1” to “If a household *i* has completed all the three activities involving energy consumption, then *EneRef*_*i*_ = 1”. Second, we changed the regression method from Ordered Probit to Ordered Logit. Third, we did a Placebo test by manufacturing a treatment variable of Energy consumption Revolution with the random selection. We randomly selected 656 farmers from 1320 samples as the counterfactual treatment group of rural energy consumption revolution, constructed a pseudo-explanatory variable (*EneRef_fake*), and used it to estimate the above benchmark model. The above process was repeated 500 times and 1000 times respectively, and the density distribution of the regression coefficient (α_xy_) of the primary explanatory variable (*EneRef_fake*) was obtained, as shown in Figure 3. This key variable, i.e., the *EneRef_fake*'s regression coefficient α_xy_, was concentrated around 0, and the 1000 random results (right) were closer to 0 than the 500 random results (left). These results indicated that all these robustness tests were passed.

## Discussion

Overall, our results show that the rural energy consumption revolution improved farmers' happiness. However, the mediation analyses show that the direct effect accounted for over 90% of the total effect; the revolution increased farmers' happiness is only through increasing leisure activities, but not through improving household income or living environment. To achieve ultimate success for energy consumption revolution to be successful, it must realize the full potential of the revolution by promoting its impact on the socioeconomic and environmental factors, so as to develop a multi-dimensional mechanism for increasing happiness.

While the rural energy consumption revolution has marginally increased farmers' happiness by increasing leisure activities, its negative impact on happiness for higher electricity expenditure is also significant especially to rural low-income households. In other words, the revolution is a double-edged sword, not only to increase leisure activities by liberating people from daily chores, but also to raise people's electricity expenditure, which leads to additional financial burden for rural low-income households. Therefore, when promoting the energy consumption revolution in rural areas, it is important to regulate electricity and gas prices, so as to guarantee that they are affordable for farmers. In addition, the energy consumption revolution has yet to improve the rural living environment, which reflects a low energy utilization rate (i.e., incomplete energy upgrades) in rural China.

Our results also show that geographic region and household income level moderated the relationship between energy consumption revolution and farmers' happiness. It is found that rural residents of less-developed Western China or low-income households were more likely to respond to the energy consumption revolution. Besides, rural residents of Central-Eastern China, where are more-developed, including middle and high-income households, pay more attention to increasing leisure activities brought by the energy consumption revolution, while rural residents of Western China or low-income households were more sensitive to the spending of electricity use.

Results from our study are meaningful for policy implications. First, it is difficult to promote the rural energy consumption revolution only as a national policy. It is important to act synergistically, by implementing or integrating it with other relevant national policies, such as policy for ‘Pollution Prevention and Abatement' (35) and ‘Poverty Reduction' (36). It is observed that only 23.5% of the rural households have completed energy upgrade, it shows that the revolution did encounter obstacles in rural China. Lack of a multi-dimensional mechanism for improving farmers' happiness is the main reason for it. Therefore, it is necessary to synergistically implement the rural energy consumption revolution with the national targeted poverty alleviation policy as well as the national pollution prevention and control policy, forming a multi-pronged strategy that simultaneously targets the socioeconomic and environmental factors associated with farmers' happiness.

Second, from the perspectives of both equity and efficiency, to introduce an energy policy that favors the poor Western regions and low-income households are important for promoting the rural energy consumption revolution. In terms of equity, “Energy Poverty Reduction” (37) is an important part of the national target of poverty reduction policy. Our results suggest that promoting the energy consumption revolution in Western China and low-income households can increase happiness. As to efficiency, the poor Western China and low-income households should be paid more attention in the energy consumption revolution. The benefits of the new energy policy, including national investments in energy infrastructure, subsidies for terminal equipment (e.g., heater, refrigerator, and air conditioner), especially price regulations related energy use, should be introduced firstly to the poor rural areas, so that farmers' happiness can be increased. In return, it will further facilitate the rural energy consumption revolution.

## Data Availability Statement

Publicly available CGSS datasets were analyzed for this study, which can be found here http://cgss.ruc.edu.cn/. Further inquiries can be directed to the corresponding author.

## Author Contributions

ZX conceived the model and analyzed results. RG collected the data. ZX and RG wrote the manuscript together. All authors contributed to the article and approved the submitted version.

## Funding

This study was supported by the China National Philosophy and Social Science Foundation in 2021: Research on the dynamic mechanism and realization path of urbanization with county as an important carrier (21BJY017).

## Conflict of Interest

The authors declare that the research was conducted in the absence of any commercial or financial relationships that could be construed as a potential conflict of interest.

## Publisher's Note

All claims expressed in this article are solely those of the authors and do not necessarily represent those of their affiliated organizations, or those of the publisher, the editors and the reviewers. Any product that may be evaluated in this article, or claim that may be made by its manufacturer, is not guaranteed or endorsed by the publisher.
